# When the local meets the global: models and challenges of primate conservation in the Fazao-Malfakassa National Park, Togo

**DOI:** 10.3897/zookeys.1276.169771

**Published:** 2026-04-07

**Authors:** Noundja Liyabin, Folega Fousseni, Polo-Akpisso Aniko, Beckline Mukete, Eve Bohnett, Chabi Djagoun, Segniagbeto Hoinsoude Gabriel, Wala Kperkouma, Komlan Batawila

**Affiliations:** 1 Laboratory of Botany and Plant Ecology; Faculty of Sciences, University of Lomé, BP 1515, Lomé, Togo Laboratory of Botany and Plant Ecology; Faculty of Sciences, University of Lomé Lomé Togo; 2 United Nations Development Programme (UNDP), 40, Avenue des Nations Unies BP. 911 Lomé, Togo Laboratory of Ecology and Ecotoxicology, Faculty of Sciences, University of Lomé Lomé Togo; 3 Center for Forests and Climate Change, Agrosystems Group, Box 76 Tiko, Cameroon Laboratory of Applied Ecology, Faculty of Agronomy Sciences, University of Abomey Calavi Cotonou Benin; 4 Fulbright Scholar, Swarthmore College, University of Massachusetts Boston, University of Michigan, Florida, USA United Nations Development Programme (UNDP) Lomé Togo; 5 Laboratory of Applied Ecology, Faculty of Agronomy Sciences, University of Abomey Calavi, Cotonou, Benin Center for Forests and Climate Change, Agrosystems Group Tiko Cameroon; 6 Laboratory of Ecology and Ecotoxicology, Faculty of Sciences, University of Lomé, BP 1515, Lomé, Togo Fulbright Scholar, Swarthmore College, University of Massachusetts Boston, University of Michigan Florida United States of America

**Keywords:** Conservation status, ethnoprimatology, Fazao-Malfakassa National Park, human-primate interactions, local ecological knowledge, Togo

## Abstract

Protected areas are created to serve two major objectives, which include the securing of ecosystem services and the conservation of biodiversity. However, the status of primates around the Fazao-Malfakassa National Park, which is the last refuge for threatened primates in Togo, is a call for concern. Using a questionnaire survey with a semi-structured interview protocol, this study examines primate diversity and its relationship with surrounding communities. Of the 12 primate species identified through community interviews, *Erythrocebus patas* was the most frequently cited (23.18% of citations), followed by *Cercopithecus mona* (14.29%). The species assemblage included the endangered *Colobus vellerosus* and the locally extinct or extremely rare *Pan troglodytes verus*. Analysis of human-primate relationships revealed that local communities utilize primates primarily as a food source (45.45% of respondents), for cultural representations (31.82%), and for religious purposes (18.18%) (percentages calculated from multiple-response questionnaires). The study also found three endogenous primate conservation and management principles that focus on the control of crop destruction and adherence to local cultural and religious norms. Due to this complex relationship between local populations and primates, it is necessary to adapt integrated approaches that combine monitoring efforts, awareness campaigns, law enforcement, and ecotourism development while prioritizing primate conservation and ecosystem functions.

## Introduction

Biodiversity conservation is one of the major challenges of the 21^st^ century. Among the many facets of this crisis, primates, an emblematic and ecologically vital taxonomic group, are particularly at risk. According to the International Union for Conservation of Nature (IUCN), more than half of all primate species are classified as Vulnerable, Endangered, or Critically Endangered (Fernández et al. 2022). This alarming situation is primarily driven by anthropogenic factors, notably habitat loss and fragmentation, illegal hunting, and pressures induced by climate change. Agricultural encroachment, logging, and uncontrolled grazing degrade primate habitats while simultaneously exacerbating human-wildlife conflicts (Beckline et al. 2018).

In the face of these threats, protected areas (PAs) have been established as the cornerstone of global conservation strategies (Cazalis et al. 2020). Designed to safeguard ecosystems and provide a refuge from direct human pressures (Geldmann et al. 2019), PAs have evolved into powerful political and socio-economic instruments aimed at enhancing both ecological and social resilience (Madzous et al. 2021; Masullo et al. 2019). In response to global commitments such as the Aichi Targets, their number and coverage have increased significantly in recent decades (Molina et al. 2019; Maxwell et al. 2020). Paradoxically, the continued expansion of the human footprint has led to ongoing degradation and fragmentation of these protected landscapes, thereby making conservation goals increasingly difficult to achieve (Geldmann et al. 2019; Liyabin et al. 2025b). Traditional conservation models, which are often strictly regulatory in nature, are showing their limitations, struggling to adapt to mounting socio-ecological pressures (Fousseni et al. 2012; Geldmann et al. 2019; Yuan et al. 2024). For primates, which usually live near humans, the mere delineation of a protected zone, regardless of its legal status or size, is no longer sufficient to ensure their long-term survival (Mollier 2023; Yuan et al. 2024).

In Togo, the Fazao-Malfakassa National Park (FMNP) perfectly illustrates this tension between conservation imperatives and human pressures. As the largest protected area in the country, it holds significant ecological and ecotourism potential, especially due to its mountainous terrain, which supports high biological diversity (Atsri et al. 2018a). Nevertheless, these assets have not been enough to counteract growing threats linked to agricultural expansion and habitat fragmentation (Atsri et al. 2018b; Tchamie and Lare 2014). The relative failure of the historical management model, characterized by regulatory and military approaches, has been further exacerbated by years of sociopolitical instability. In response, a new management approach has been initiated through the Franz Weber Foundation (FWF), aiming to strike a balance between conservation and local development. However, due to multiple constraints, the implementation of a fully collaborative management framework remains challenging, leading to renewed anthropogenic pressures and escalating human–wildlife conflicts, particularly with primates (Atsri et al. 2020; Wala et al. 2012; Woegan et al. 2013).

In this context, primate conservation has become a central issue in the management of FMNP (Agbessi et al. 2017; Campbell et al. 2007; Noundja et al. 2025). To date, primatological research in Togo has primarily focused on diversity and distribution inventories (Agbessi et al. 2024; Ségniagbeto et al. 2018). Yet, cultural, social, and economic factors among local populations play a decisive role in shaping attitudes and behaviors toward primates.

Ethnoprimatology, an interdisciplinary field that combines primatology and the social sciences (Fuentes 2012), provides a relevant framework for addressing this complexity. Drawing on local ecological knowledge, this study aims to inform conservation strategies and assess opportunities for better integration of communities into park governance.

The overall objective of this study is to contribute to more effective primate conservation in FMNP by adopting an ethnoprimatological perspective that integrates local ecological knowledge with conservation science. Specifically, the study aims to: (i) document local perceptions of primate diversity and distribution, and identify the socio-cultural and anthropogenic factors shaping these perceptions; (ii) characterize the nature and dynamics of human–primate interactions, including conflicts, coexistence practices, and cultural representations; and (iii) evaluate how current park management strategies influence local attitudes toward primates and assess community-based conservation initiatives that could enhance human–primate coexistence.

## Materials and methods

### Study area

**Figure 1.**
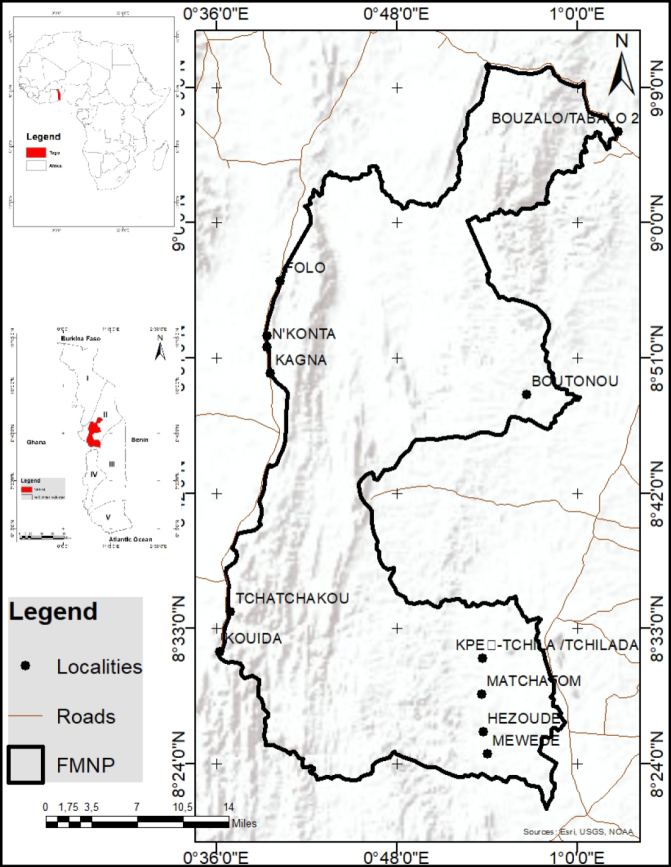
Location of Fazao-Malfakassa National Park in central Togo, West Africa.

Fazao-Malfakassa National Park (FMNP) is one of Togo’s most important protected areas. Established in 1975, it resulted from the merger of the Fazao Forest Reserve (162,000 ha) and the Malfakassa Forest Reserve (30,000 ha) (Woegan et al. 2013). Located in the heart of the Atakora Range, the park extends approximately between latitudes 8°30' and 9°30'N and longitudes 0°45' and 1°15'E (Fig. 1). The core study area centers on Mount Fazao (861 m) and Mount Malfakassa (713 m) (Atsri et al. 2020). The park is located in the central administrative region, at the transition between ecological zones 2 and 3, characterized by a predominantly open forest, dry forest, riverine forest, and savanna-type vegetation (MPTHU 2001; Woegan 2011). The region experiences a unimodal rainfall regime, with annual precipitation ranging from 1,200 to 1,500 mm, and an average temperature of 25 °C during the rainy season. Local communities, mainly comprising the Tem, Agnanga, Adélé, Bassar, and Kabyè ethnic groups, practice subsistence farming, favoring the clearing of savannah trees for their fertility.

However, the park is increasingly subject to human pressures such as poaching, logging, grazing, and the harvesting of non-timber forest products. Recognized on UNESCO’s World Heritage Tentative List, the FMNP stands out for its rich ecosystem services, both in terms of wildlife conservation and ecotourism development (UNESCO 2013). The Park is an important refuge for wildlife diversity, making it an outstanding example of in situ conservation success for primates in Togo. It is therefore the best-preserved protected area in Togo and has significant ecotourism potential, where a diversity of wild fauna such as elephants, buffalo, and several species of primate can still be observed, as well as being the only ecosystem where seed bananas (*Ensete gilletii*) grow in Togo (MERF-Togo 2020).

### Sampling of survey areas

We apply a hierarchical sampling design to determine both the eligible villages and the specific local actors to be surveyed. For the spatial delimitation of communities, a geospatial overlay was performed using the regulatory shapefile of the protected area boundaries and the national census layer of localities and their population based on the Fourth General Population and Housing Census data, within the QGIS (Quantum GIS) environment.

A 500-meter buffer zone was created around the park’s boundaries, based on the hypothesis that this represents the maximum range of primates’ excursions beyond their core habitats under natural conditions (Naughton‐Treves 1998, 2002; Zoffoun et al. 2019). All villages, hamlets, and agricultural settlements located within this buffer zone and with a recorded population exceeding 100 individuals were retained. Following this procedure, twelve villages located either within or directly adjacent to the park were selected as sampling sites.

Initial contact was established by telephone with village chiefs and the chairpersons of local development committees (Village Development Committees – VDCs), through the facilitation of the park’s head warden and forest patrol officers. These preliminary consultations aimed to provide local authorities with comprehensive information on the project’s objectives and to agree on a consensual timetable for the identification and mobilization of target participants. Verbal FPIC was obtained from all participants, with anonymity ensured through aggregated reporting and no individual identifiers. No formal IRB was required under Togolese regulations, but ethical guidelines from the International Primatological Society were followed.

The sampling of participants within each selected village was purposive, focusing on individuals with relevant ecological knowledge or direct interactions with the forest landscape. This included agro-pastoralists, hunters, forest guides, traditional leaders, and other actors involved in natural resource use (Bloomfield et al. 2020). Stratification was applied to ensure representation across occupational groups directly affected by or contributing to human–primate interfaces.

### Ethical approach and consent

An official research authorization (No. 0402/MERF/SG/DRF dated 29 October 2024) was obtained from the Ministry of Environment and Forest Resources of Togo, ensuring compliance with national ethical and legal standards. The protocol involved a rigorous process of obtaining Free, Prior, and Informed Consent (FPIC) beforehand. All concerned communities were formally informed of the study’s objectives, duration, and the intended scientific use of the collected information through their local leaders (village chiefs and village development committees). This step was facilitated by the park Curator and forest Rangers, whose involvement was essential in securing local support, establishing trust, and obtaining verbal informed consent.

### Sampling and participant selection

Participants in each village were selected using purposive (non-probability) sampling, targeting individuals with relevant ecological knowledge or those who have direct interactions with the forest landscape and wildlife (Noundja et al. 2025; Naughton‐Treves 2002). To ensure adequate representation of different perspectives and to minimize biases, stratification was applied based on socio-professional groups directly affected by or contributing to human-primate interactions. Stratification categories were identified with the help of local leaders, aiming for diverse profiles and including farmers, hunters/poachers, forest guides, traditional leaders, beekeepers, and artisanal loggers. The objective was to include a minimum of 15 participants per discussion group, with a target of 20–25 to enrich the dialogue. Aware that perceptions and knowledge can significantly differ between men and women due to their distinct roles and interactions with the environment (Kama 2019), efforts were made to include women in the discussion groups. However, due to local socio-cultural constraints and availability, female participation was limited, resulting in a male predominance (84.8% men). Female participation was limited to 15.2% due to socio-cultural constraints (e.g., gender roles in agricultural activities), potentially biasing results toward male-dominated perspectives on hunting and conflicts. Future studies should employ targeted recruitment to balance representation.

### Conduct of ethnoprimatological surveys

Twelve (12) focus group discussions were conducted, one in each selected community, with an average of 23 participants per locality. Each session lasted approximately 1 hour and 20 minutes and was guided by a semi-structured interview protocol inspired by a review of specialized ethnoprimatological literature (Gandiwa 2012). Interviews were conducted in local languages, and the data were systematically recorded using digital forms on KoboCollect.

The questionnaire was designed to gather extensive information on local knowledge and perceptions of qualitative trends in primate populations within FMNP, with a focus on observation frequency, interaction diversity, and conservation potential. The interviews explored a wide range of topics, including local knowledge of primate species diversity, observed behaviors and interspecific interactions, symbolic and cultural representations of primates, reported conflicts or coexistence practices, habitat characteristics, and perceived ecological threats to primates and their environments. Primate species identification was based on a photographic recognition method using a visual board of confirmed or potentially present primate species in Togo, grounded in taxonomic references, to validate vernacular names and visual recognition. Best-practice methodologies for defining the context of human-wildlife conflict were applied to ensure scientific transparency and reproducibility in ethnoprimatology, inspired by the conventional literature on conservation conflicts (Baynham-Herd et al. 2018; Haddaway et al. 2015).

### Data analysis and processing

Ecological parameters assessed included: local knowledge of primate species diversity within the park, perceived species-specific vulnerability levels, and habitat characterizations. These variables were analyzed using the percentage frequency of species citations, calculated from aggregated interview data. Descriptive statistics and contingency tables were generated using standard methods and Dynamic cross-tabulations, adapted according to the type and structure of each variable. To assign conservation status to each cited primate species, IUCN Red List data were systematically consulted (IUCN 2026), ensuring alignment between local knowledge and international conservation frameworks.

The local conservation status of a species is assessed over a ten-year period (from 2015) using specific indicators: abundance (encompassing both solitary individuals and conspecific groups), citation frequency, distribution, and demographic trend. The determination of the local status of species vulnerability is inspired by (Agbani et al. 2018), by calculating the frequencies and average order of specific citations according to the hypothesis that the least vulnerable species are the most freely recognized. Similarly, the least threatened species are those with a more stable or increasing demographic trend (Albuquerque et al. 2014), whereas frequency of encounter defines relative abundance, and frequently encountered species are assumed to have relatively low vulnerability. Local status on the basis of qualitative demographic data is scaled from 1 to 5, based on global assessments of the number of individuals encountered in a year, compared with specific IUCN status (Table 1).

**Table 1.** Scoring matrix for determining local conservation status of primates (frequency + order of citation + abundance + trend = total score / local status).

Score	Citation frequency	Identification order	Abundance	Demographic trend	Total score	Local status
5	> 75%	1–2 (easily identified)	Very abundant (≥10 obs./month)	Stable or increasing	18-20	Least Concern (LC)
4	51-75%	3-4	Fairly common (3-9 obs./month)	Slight decline	14-17	Near Threatened (NT)
3	26-50%	5-6	Rare (1-2 obs./month)	Moderate decline	10-13	Vulnerable (VU)
2	10-25%	7-8	Very rare (≤1 obs./year)	Severe decline	6-9	Endangered (EN)
1	< 10%	9-10 (poorly known)	Locally extinct (0 obs. in last 5 years)	Confirmed extinction	1-5	Extinct (EX)

Thresholds were adapted from (Agbani et al. 2018) and calibrated to local contexts via pilot interviews, e.g., ≥ 10 observations/month for ‘Very abundant’ based on community-reported encounter rates.

### Inferential statistical analyses

Beyond descriptive analyses, inferential statistical tests were conducted to examine ecological relationships and potential correlations among variables. All statistical analyses were performed using R software (version 4.0.2) (Wickham et al. 2020), utilizing relevant packages for multivariate analysis and data visualization.

A comparison was made of the citation frequencies of species recognized and considered important by local communities, according to villages and sociolinguistic groups. An Analysis of Variance (ANOVA) or a Kruskal-Wallis test was used depending on whether the assumptions of the application (normality, homoscedasticity) were met, or when the non-parametric nature of the data justified it (Agbani et al. 2018; Dytham 2011). Pearson’s chi-square test and Fisher’s exact test were used to assess collinearity between species perception data and sociocultural or environmental variables. Particular attention was paid to pairs of variables showing high correlation coefficients (r > 0.7) or Variance Inflation Factors (VIF) exceeding 5, in order to avoid multicollinearity issues in multivariate models. Strongly collinear variables were identified, and where necessary, only one variable from each collinear group was retained for the final models, or dimensionality reduction methods were considered.

Several probabilistic statistical models were tested to identify key determinants of the local vulnerability level of primates. The models considered included multivariate regression models with variable selection, including stepwise selection based on Akaike Information Criterion (AIC), generalized linear models (GLM), and mixed models (to account for the hierarchical structure of the data, e.g., villages nested within regions). The final model choice was guided by a combination of statistical criteria and the model’s compliance with underlying assumptions: (AIC) to compare model quality, Residual Deviance, and Global Model Performance Test (Nagelkerke’s Pseudo-R^2^). An R^2^ of 0.374 was considered acceptable to good models in social and ecological sciences, given the complexity of human perception factors.

For all these analyses, diagnostic plots (residuals vs fitted values, Q-Q plots of residuals, scale-location, residuals vs leverage) were systematically examined to check model assumptions (linearity, homoscedasticity, normality of residuals, absence of influential points). The ordinal regression model, fitted using the Proportional Odds Logistic Regression in R software with the function (*polr()*) from the MASS package, showed the best overall quality and fit to the assumptions, particularly considering the ordinal nature of the response variable and managing the identified collinearities. This model was therefore selected for the analysis of factors influencing the conservation status of primates in FMNP. Predictor factors included: habitat types, threats, perceived value (value_percus), local perspectives, and local conservation strategies (locale_strategies).

To assess the statistical significance of all predictor factors included in the model, two-way ANOVA tests were conducted to evaluate their effect on the perception of conservation status (p < 0.05). To enhance the interpretation of complex interactions among species, habitat types, vulnerability categories, and perceived threats, Sankey diagrams were generated. These diagrams were constructed by aggregating citation frequency data and perceived associations among variables, thereby illustrating information flows and interconnections.

## Results

### Primate diversity and conservation status of primates in FMNP

Local communities around FMNP recognized 12 primate species. Based on citation frequency across all focus group discussions, *Erythrocebus patas* (Schreber, 1775) was the most frequently mentioned species, accounting for 23.18% of total citations, followed by *Cercopithecus mona* (Schreber, 1774) (19.05%). *Papio anubis* (Lesson, 1827) and *Galago senegalensis* (Geoffroy, 1796) each accounted for 14% of citations.

**Figure 2.**
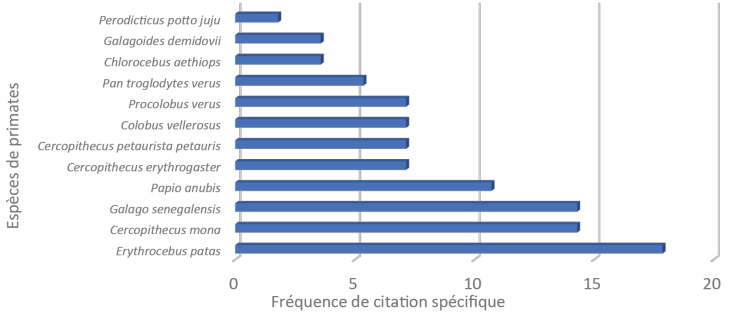
Primate species citation frequency around Fazao-Malfakassa Park.

Primate species richness varied significantly depending on several predictors. The most influential factors were anthropogenic and conservation-related variables (specifically, threat levels and IUCN Red List status), local population status, and local environmental variability (β < 0.2) (Fig. 2).

**Figure 3.**
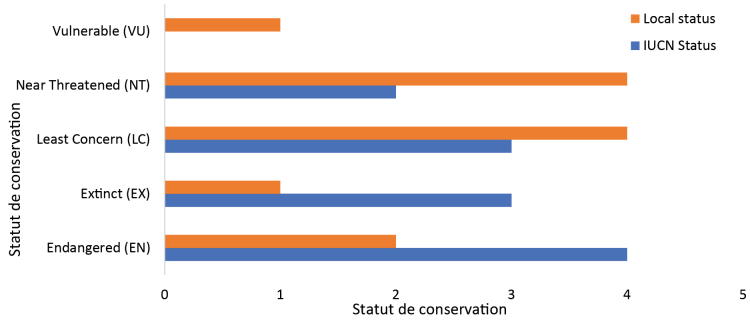
Comparative diagram of IUCN and local conservation statuses of primates.

Seven primate species, representing 58.33% of those reported around FMNP, are classified in major concern categories. These include three species listed as Vulnerable (VU), three as Extinct (EX), and one as Endangered (EN). The species currently considered locally extinct are *Cercopithecus erythrogaster* (Gray, 1866), *Cercopithecus petaurista petaurista* (Schreber, 1774), and *Pan troglodytes verus* (Schwarz, 1934), while *Colobus vellerosus* (I. Geoffroy, 1834) is critically endangered at the local level. Only five species fall under the lower concern categories, including *Cercopithecus mona*, *Chlorocebus aethiops* (Linnaeus, 1758), and *Papio anubis*, which are classified as Near Threatened (NT), and *Erythrocebus patas* and *Galago senegalensis*, which are categorized as Least Concern (LC) (see Fig. 3). However, according to the IUCN Red List, the Least Concern species are the most represented overall, with four species (33%) being in the Near Threatened (NT) and Least Concern (LC) categories. The Kruskal-Wallis test indicates a highly significant statistical difference between IUCN conservation statuses across the different local status classes (p-value < 0.0001).

### Ecological determinants of diversity and local conservation status of primates

**Figure 4.**
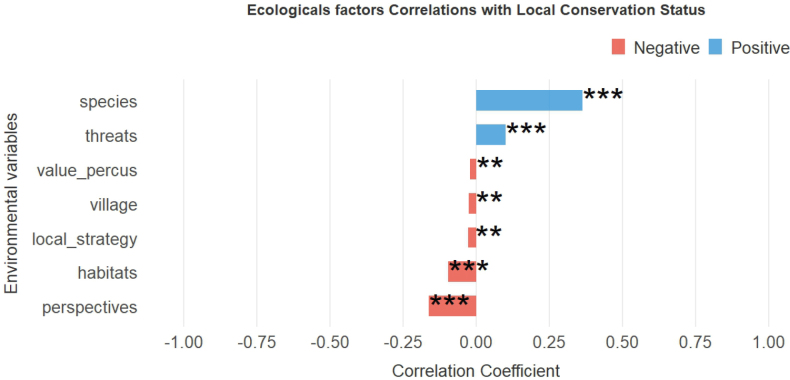
Variation in correlation coefficients and significance between ecological variables and primate species diversity. Legend: ***p < 0.001; **p < 0.01; *p < 0.05.

Seven ecological factors have a statistically significant influence on the local conservation status of primates in FMNP. The analysis of local conservation factors reveals that species diversity and the variability of threats are both highly significantly correlated (p < 0.001). In contrast, habitat diversity, and conservation perspectives (i.e., proposed conservation actions) have strong but negative ecological influences on primate conservation. Although only weakly correlated, the factors of perceived value diversity, village proximity, and local strategies also significantly influence the local conservation status of primate species (Fig. 4).

Regarding threats, the most significant are habitat loss (39.7%) and wildfires (31.4%), followed by agricultural expansion (14.6%). Secondary threats include poaching, overgrazing, and the withdrawal of the Franz Weber Foundation (FWF). The departure of the Foundation corresponds to a reduction in the capacity to protect, monitor, and secure the boundaries of the protected area. These ecological threats clearly explain the level of primate vulnerability. The most threatened species were those with concerning statuses, particularly those already locally extinct (EX) (14.2%) or classified as Endangered (EN) (13.1%).

In terms of habitat diversity, forest ecosystems are recognized as natural habitats for primates. These are further subdivided into plain forests, including gallery forests (34.37%), mountainous ecosystems (31.25%), and agroforests (18.75%). The main threats to these habitats are listed in Fig. 6.

**Figure 5.**
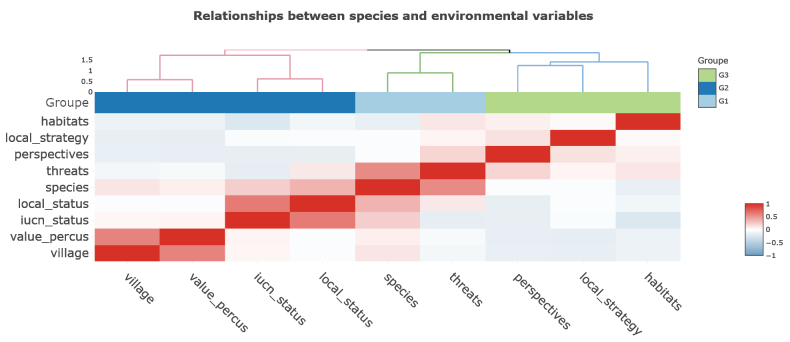
Statistical correlation matrix between groups of fundamental ecological variables describing the diversity and conservation status of primate species in FMNP.

**Figure 6.**
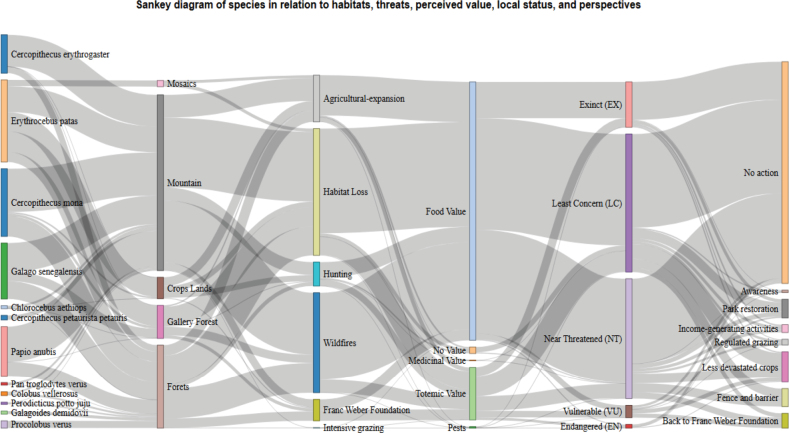
Sankey diagram illustrating the interconnections between primate species, their associated habitats, and the specific anthropogenic threats they face in the Fazao-Malfakassa National Park.

Primates are primarily perceived as a food resource (80.9%), followed by cultural representations such as totems and taboos (16.5%), as species without specific value (2.0%), as pests (0.4%), and, to a lesser extent, as medicinal resources (0.2%).

Some conservation strategies are being considered to improve coexistence and conservation of primates (Fig. 5), including park restoration (69.39%), cultivation of less-devastating crops (9.50%), construction of physical barriers (5.6%), reinstatement of the delegated management by the Franz Weber Foundation (FWF) (4.6%), implementation of alternative income-generating activities (2.46%), regulation of grazing (1.83%), and awareness campaigns (0.74%). However, 5.84% of the community surveyed believe that no action would have a real impact on primate conservation.

Beyond these perspectives, effective management measures for crop depredation also represent ecologically significant factors influencing primate conservation (Fig. 5). The most commonly employed strategies include regular human surveillance (sometimes with guard dogs) (27.33%), installation of scarecrows (27.06%), cultivation of less attractive crops (18.60%), deterrent hunting (17.07%), use of fire and smoke (6.27%), and finally, the use of sound deterrent devices (3.67%).

### Ecological conservation performance

Multivariate statistical analyses indicate that poaching (β = -0.12, p-value < 0.001), agricultural expansion (β = -0.08, p-value = 0.003), and vegetation fires (β = -0.05, p-value = 0.021) are the most significant threats associated with species vulnerability status. In contrast, field patrolling (β = +0.21) and less-preferred alternative crops (β = +0.15) appear to be highly effective adaptive mitigation strategies against crop devastation caused by primates. A cross-analysis of specific threats and local management measures for human–primate interactions reveals strong correlations, particularly between poaching and regular field guarding (β = +0.21), and between agricultural expansion and alternative crops (β = +0.15).

An in-depth descriptive analysis of key ecological factors indicates a strong statistical correlation between IUCN status and species diversity. Species classified as Endangered or Vulnerable (EN/VU) tend to be more exposed to habitat loss threats (R^2^ = 0.893, p < 0.001).

### Primate conservation models in Fazao-Malfakassa National Park

The analysis revealed four primary habitat types, ordered by decreasing relative coverage: mountain ecosystems (55%), forests (26%), gallery forests (10.3%), and mosaics of anthropized habitat fragments (6.7%). The model indicates that the most vulnerable species tend to prefer dense forest habitats (β = +3.68; t = 30.98) and mountainous areas (β = +3.90; t = 33.25), whereas habitats more exposed to wildfires (β = –1.74) and intensive grazing (β = –1.84) are typically occupied by species of lesser conservation concern. Promising avenues for effective primate conservation actions include the return of the FWF (β = +1.68; t = –5.96), as well as the installation of physical barriers such as fences (β = –1.38; t = –5.73). Primate species with local totemic cultural significance are mostly among the highly vulnerable species (β = +0.69), in contrast to species without such cultural value (β = –2.55). In the model, acoustic deterrents emerge as the most effective strategies to mitigate human–primate conflicts (β = –0.36). This deterrent strategy is associated with a slight decrease in perceived risk, indicating a certain level of trust in its effectiveness.

### Discrimination of primate diversity based on ecological determinants

Three ecological groups of primate species were identified (Table 2) (Average Silhouette = 0.72):

**Table 2.** **C**lassification of primate species into ecological groups.

Cluster (number of species)	Characteristics	Representative species
1 (*n* = 4)	High threat, active strategies	*C. vellerosus*, *P. verus*
2 (*n* = 5)	Low threat, stable status	*E. patas*, *G. senegalensis*
3 (*n* = 3)	Critical threats, weak strategies	*C. erythrogaster*

- Group G1 (*n* = 4 species) includes highly threatened species with concerning conservation status (Endangered and Vulnerable), which are poorly protected by local measures and strategies (*C. vellerosus*, *P. verus*).
- Group G2 (*n* = 5 species) consists of species that are better adapted to threats or only slightly threatened, with a more stable status and benefiting from more appropriate conservation actions (*E. patas*, *G. senegalensis*).
- Group G3 (*n* = 3 species) includes very highly threatened and highly vulnerable species for which existing strategies are poorly adapted (*Cercopithecus erythrogaster*).

## Discussion

This exploratory ethnoprimatological study investigates local perceptions of primate conservation status and the socio-ecological factors influencing these perceptions around FMNP.

The observed primate diversity was dominated by *E. patas*, *C. mona*, and *G. senegalensis*. The species richness reported in this study (12 species) differs from previous inventories in Togolese Protected Areas, which documented eight or nine species (Agbessi et al. 2024; Agbessi et al. 2017; Amori et al. 2016; Segniagbeto et al. 2017). This disparity warrants careful examination. Three species *Pan troglodytes verus*, *Cercopithecus petaurista petaurista*, and *Cercopithecus erythrogaster* warrant particular discussion. *Pan troglodytes verus* was reported by communities as historically present but now locally extinct, corroborating recent studies that found no evidence of chimpanzee persistence in Togo (Segniagbeto et al. 2017). For *C. petaurista petaurista* and *C. erythrogaster*, their reported presence requires cautious interpretation. *C. erythrogaster* is confined to the Togodo Protected area border region (IUCN 2026).

These observations raise important questions on the potential misidentification due to reliance on photographic recognition alone, the possible range shifts or undocumented historical distributions, or the persistence of very small, isolated populations unknown to science. Rather than dismissing community reports as errors, we interpret them as hypotheses requiring further investigation through complementary methods such as camera trapping, genetic analysis, and targeted field surveys.

Nonetheless, this study highlights the importance of Fazao-Malfakassa Park as a protected area with ecosystemic diversity that serves as an essential refuge for primate species diversity and habitats within the current network of Protected Areas in West Africa. Indeed, according to (Thool 2020), the park’s topographic variation provides a plethora of microhabitats that allow different primate species to access refuges and food resources suited to their ecological preferences.

Furthermore, the chimpanzee, *P. troglodytes verus* (EX) (IUCN 2026), has been reported by local communities as locally extinct. This confirms results from numerous recent studies, which found no evidence of chimpanzee (*P. troglodytes verus*) presence in Togo (Segniagbeto et al. 2017). One of the most striking findings of this study is the significant divergence between locally perceived primate conservation status and the official classifications of the International Union for Conservation of Nature (IUCN). While the IUCN provides a global assessment based on rigorous scientific criteria, local communities, through their daily interactions with wildlife, develop a nuanced understanding of species abundance, distribution, and demographic trends at finer spatial and temporal scales (da Silva Costa et al. 2023). This divergence may be explained by several factors, including differences in scale, specific contextual knowledge not captured by global assessments, or perception biases related to direct (positive or negative) interactions with primates (Tran et al. 2025). The local classification of some species as “Extinct” (EX) or locally Endangered, whereas they may hold a less critical status on the IUCN scale, underscores the urgency for targeted local conservation actions (Koné et al. 2020; Oates et al. 2020; Wallis 2023).

Local communities identify habitat loss caused by overgrazing, expansive agriculture, poaching, vegetation fires, and the departure of the Franz Weber Foundation as the main threats making primates vulnerable in situ. These threats are positively correlated with more concerning conservation statuses (Locally = 14.2% EX and 13.1% EN), highlighting the direct impact of anthropogenic pressures on primate populations (Wallis 2023). According to (Oates et al. 2020), several monkey species in West African forests, such as *Procolobus verus*, are widely hunted to supply meat for local family consumption. Similar trends have been observed regionally across West Africa (Angwafo et al. 2019; Fernández et al. 2022). For (Bamba et al. 2017; Bene et al. 2012), the vulnerability of primates such as the red colobus (*Piliocolobus badius*) is rather species-specific and linked to body size.

In response to the wide range of threats, communities neighboring FMNP envision the implementation of certain conservation actions. These actions include controlling grazing, adopting less attractive crops, restoring degraded habitats, the return of the FWF, and the construction of physical barriers. These conservation prospects highlight the crucial role of community engagement and local initiatives in shaping perceptions of conservation effectiveness (Killion et al. 2021). Similarly, the correlation between perceived value and local human-primate conflict management strategies relative to conservation status suggests their more nuanced influence, interpreted as recognition of the cultural importance of species, especially those in decline (Setchell et al. 2017; Sharma et al. 2021).

Primate distribution is determined by the structural and topographic characteristics of their ecosystems, with a strong preference for dense forest habitats and rugged mountainous topography. Model results indicate that the most vulnerable species seek refuge in dense forests and less anthropogenically pressured mountain habitats, reflecting their low adaptability to anthropogenic pressures. Similar studies (Dewanou et al. 2023; Orimaye 2024) have shown that species such as *C. petaurista* and *P. verus* show a strong affinity for mountainous regions and dense primary forests. Meanwhile, species with greater ecological flexibility, like *E. patas*, exhibit a broader habitat range, including marginal and human-modified areas. According to some authors (Kalbitzer and Chapman 2018), habitat selection may not be a preference but rather a constraint driving species to refugial habitats less influenced by humans and providing natural protection from predators. However, many primate species adapt to anthropogenic threats in their habitats as a strategic response (Bryson-Morrison et al. 2017; McLennan et al. 2017). This adaptation or adaptive flexibility presents an ecological challenge (Viallard et al. 2024; Villain 2017).

The three endogenous management principles, including crop depredation control, totems and taboos, and religious prohibitions and their influences on the perception of conservation status, help target conservation efforts (Bamba et al. 2017). Also, the effectiveness of acoustic deterrents as deterrent tools aligns with observations by (Galán-Acedo et al. 2021) on non-lethal methods to reduce human-primate conflicts. Thus, biodiversity conservation initiatives should be designed collaboratively with communities, considering their perspectives and needs to ensure their buy-in and long-term effectiveness (Pooley et al. 2021). However, it is crucial to account for perception variability among communities in planning local management measures (Stafford et al. 2016).

The edges of Protected Areas, such as those of FMNP, present a particular environmental challenge, as these zones concentrate anthropogenic threats and serve as preferred ecosystems for certain primate species. The classification of primates into three ecological groups reflects gradients of vulnerability and effectiveness of protective measures. This classification implies the implementation of differentiated conservation actions. The group of highly threatened but poorly protected species requires urgent interventions (IUCN 2026). Group 2 comprises more resilient species considered stable due to their ecological plasticity (Bersacola et al. 2023). Conversely, the composition of group 3 translates to species in danger requiring urgent interventions and stronger management measures (Koné et al. 2020). Therefore, for FMNP, there is an urgent need for differentiated habitat and species management models combining strict protection of favorable habitats, mitigation of anthropogenic pressures, and strengthening culturally accepted strategies (Braga Gomes et al. 2024). More effective, sustainable management models are necessary for integrated primate conservation, along with addressing the critical impact of climate change on these mountainous ecosystems.

## Conclusions

This study highlights the biological diversity of primates in FMNP and their interactions with local communities. Twelve primate species were recognized by the communities living around the park, with *E. patas* and *C. mona* being the most frequently identified. A close relationship emerges between the locally known conservation status of primate species and the complexity of ecological factors. Seven primate species reported around FMNP are classified in categories of major conservation concern, including three Vulnerable (VU), three Extinct (EX), and one Endangered (EN) species.

However, several local strategies and prospective safeguarding actions—rooted in beliefs and cultural practices—aim to support an endogenous form of primate conservation. The ordinal regression model reveals that local perceptions of primate conservation status are a complex construct, significantly shaped by habitat ecological characteristics, perceived threats, cultural values, and the perceived effectiveness of conservation strategies.

The results emphasize that ecological factors are filtered through a sociocultural lens. The strong influence of habitat types, attributed species values, and community-based evaluations of management strategies provides crucial insights. These findings underscore the need to integrate local perceptions into the design of conservation plans to ensure their contextual relevance, social acceptance, and ultimately, their success on the ground.

Finally, the statistical discrepancy between IUCN statuses and local residents’ perceptions reflects the need to deepen methodological and comparative scientific research for an accurate assessment of primate vulnerability. Based on these findings, it will better establishing a participatory monitoring network involving trained community members to track primate populations, particularly for species with uncertain status, reinforcing co-management and implementing differentiated conservation strategies tailored to the three ecological groups identified, with urgent interventions for critically threatened species (Group 3). In parallel, strategic scientific safeguarding efforts should focus on an in-depth analysis of species-specific vulnerability risks, based on threats and levels of adaptation, within a contextually grounded approach.

## Additional information

### Use of AI

No use of AI was reported.

### Author ORCIDs

Noundja Liyabin https://orcid.org/0009-0004-2039-5641

Folega Fousseni https://orcid.org/000-0001-9097-3524

Polo-Akpisso Aniko https://orcid.org/0000-0002-2196-5456

Beckline Mukete https://orcid.org/0000-0002-8344-4015

Eve Bohnett https://orcid.org/0000-0002-1870-8897

Chabi Djagoun https://orcid.org/0000-0002-6352-2450

Segniagbeto Hoinsoude Gabriel https://orcid.org/0000-0002-4697-3671

Wala Kperkouma https://orcid.org/0000-0002-7533-6356

Komlan Batawila https://orcid.org/0000-0003-2781-3063

### Data availability

All of the data that support the findings of this study are available in the main text.
